# Multiscale Imaging Reveals the Hierarchical Organization of Fibrillin Microfibrils

**DOI:** 10.1016/j.jmb.2018.08.012

**Published:** 2018-10-19

**Authors:** Alan R.F. Godwin, Tobias Starborg, David J. Smith, Michael J. Sherratt, Alan M. Roseman, Clair Baldock

**Affiliations:** 1 Wellcome Centre for Cell-Matrix Research, School of Biological Sciences, Faculty of Biology, Medicine and Health, University of Manchester, Manchester Academic Health Science Centre, Manchester, M13 9PT, UK; 2 Division of Cell-Matrix Biology and Regenerative Medicine, School of Biological Sciences, Faculty of Biology, Medicine and Health, University of Manchester, Manchester Academic Health Science Centre, Manchester, M13 9PT, UK; 3 Division of Molecular and Cellular Function, School of Biological Sciences, Faculty of Biology, Medicine and Health, University of Manchester, Manchester Academic Health Science Centre, Manchester, M13 9PT, UK

**Keywords:** ciliary zonules, serial block-face imaging, fibril electron tomography, extracellular matrix, single-particle analysis of fibers, TGFβ, transforming growth factor-β, MFS, Marfan syndrome, WMS, Weill–Marchesani syndrome, EGF, epidermal growth factor-like, TB, TGFβ-binding, SBF-SEM, serial block-face scanning electron microscopy, TEM, transmission electron microscopy, ADAMTS, A Disintegrin and Metalloproteinase with Thrombospondin motif, FSC, Fourier shell correlation, QFDE, quick freeze deep etch

## Abstract

Fibrillin microfibrils are evolutionarily ancient, structurally complex extracellular polymers found in mammalian elastic tissues where they endow elastic properties, sequester growth factors and mediate cell signalling; thus, knowledge of their structure and organization is essential for a more complete understanding of cell function and tissue morphogenesis. By combining multiple imaging techniques, we visualize three levels of hierarchical organization of fibrillin structure ranging from micro-scale fiber bundles in the ciliary zonule to nano-scale individual microfibrils. Serial block-face scanning electron microscopy imaging suggests that bundles of zonule fibers are bound together by circumferential wrapping fibers, which is mirrored on a shorter-length scale where individual zonule fibers are interwoven by smaller fibers. Electron tomography shows that microfibril directionality varies from highly aligned and parallel, connecting to the basement membrane, to a meshwork at the zonule fiber periphery, and microfibrils within the zonule are connected by short cross-bridges, potentially formed by fibrillin-binding proteins. Three-dimensional reconstructions of negative-stain electron microscopy images of purified microfibrils confirm that fibrillin microfibrils have hollow tubular structures with defined bead and interbead regions, similar to tissue microfibrils imaged in our tomograms. These microfibrils are highly symmetrical, with an outer ring and interwoven core in the bead and four linear prongs, each accommodating a fibrillin dimer, in the interbead region. Together these data show how a single molecular building block is organized into different levels of hierarchy from microfibrils to tissue structures spanning nano- to macro-length scales. Furthermore, the application of these combined imaging approaches has wide applicability to other tissue systems.

## Highlights

• Extracellular matrix fibrillin microfibrils assemble to form ocular ligaments.
• Individual beaded fibrillin microfibrils are highly symmetric biological polymers.
• Zonule fibers are composed of aligned, organized arrays of fibrillin microfibrils.
• Bundles of zonule fibers are wrapped by large fibers providing structural support.
• Fibrillin organization shows how a single building block constructs an elastic tissue.

## Graphical Abstract

**Figure:**
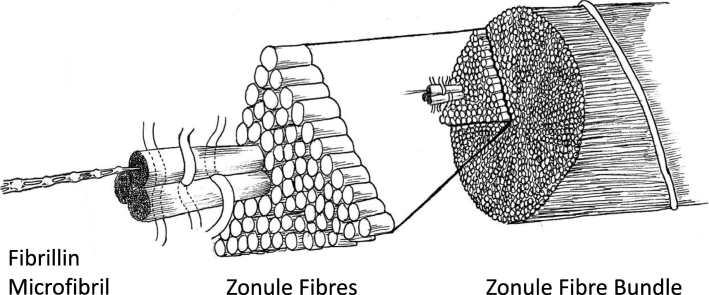

## Introduction

Fibrillins form extracellular matrix microfibrils found in many mammalian tissues such as the eye, skin, lung and vasculature where their extensible properties endow long-range elasticity [1]. They are evolutionarily ancient biological polymers that also support primitive low-pressure circulatory systems in invertebrates. Fibrillin-containing tissues such as the mammalian ciliary zonule, an ocular elastic ligament that projects radially from the lens to the ciliary muscle, is composed almost entirely of fibrillin microfibrils and is essential in transmitting force. However, we do not understand how fibrillin, a single molecular building block, is used to construct elastic tissues with nano- to macroscales of hierarchical structure to provide their unique functional properties.

Fibrillin microfibrils also play a key role in tissue homeostasis through their interaction with growth factors such as transforming growth factor-β (TGFβ) and bone morphogenetic proteins [2] and through interaction with cell surface receptors such as the integrins [3], [4] and syndecans [5]. The importance of fibrillin-1 in the function of tissues is further highlighted by fibrillin-1 mutations that cause a number of heritable connective tissue disorders termed fibrillinopathies, such as Marfan syndrome (MFS) [6] and Weill–Marchesani syndrome (WMS) [7]. MFS is characterized by overgrowth of the long bones and other skeletal abnormalities along with flexible joints, cardiovascular, lung and eye defects. Patients with WMS also have eye defects, but skeletal manifestations are almost opposite to those seen in MFS, with individuals having short stature and stiff joints.

Fibrillin-1, a ~ 350-kDa glycoprotein, comprises epidermal growth factor-like (EGF) domains interspersed by TGFβ-binding like (TB) and hybrid domains. It has been suggested that arrays of domains have a linear, rod-like structure, modeled from high-resolution structures of domain pairs and triplets [8]. However, small-angle X-ray scattering data show that in solution longer domain arrays from fibrillin and latent TGFβ-binding protein (LTBP)-1, a member of the fibrillin superfamily, are flexible and adopt non-linear conformations [9], [10]. Fibrillin assembles into microfibrils, but despite knowing the structures of some individual, pairs and triplets of domains, how individual fibrillin monomers are arranged in the microfibril is still not understood. Fibrillin microfibrils imaged in tissues have a diameter of ~ 10–12 nm [1] with a beaded appearance, and in cross section, they appear hollow with a ring of eight filaments [11], [12]. They have a mass per repeat ranging from ~ 1400 kDa, in some early fetal tissues and cell culture, to ~ 2500 kDa in adult tissues [13], [14]. This mass is consistent with up to eight fibrillin monomers per repeat. Understanding how fibrillin molecules are arranged in a microfibril is essential for understanding their assembly and locating functional sites on the microfibril such growth factor binding sites.

Fibrillin microfibrils form a number of tissue-specific higher-order structures [15]. In the medial layer of the aorta and elastic arteries, for example, elastic fibers form concentric fenestrated lamella, surrounded by circumferentially oriented smooth muscle cells [16]. A tissue where fibrillin forms bundles of microfibrils devoid of elastin is the ciliary zonule of the eye, which holds the lens in dynamic suspension and transmits force from the ciliary muscles to deform the lens in accommodation. The ciliary zonules are composed of zonular fibers [17], which contain fibrillin microfibrils [18], [19], [20], but besides these gross anatomical details, little is known of microfibril packing and organization within the ciliary zonules. However, mutations in fibrillin-1 commonly give rise to a spectrum of ocular disorders including ectopia lentis, or displacement of the lens, caused by dysfunction of the ciliary zonules [21]. Most patients with MFS or WMS have ectopia lentis [22], making this an important system to study to understand how fibrillin mutation can give rise to this pathology. Furthermore, the zonules are an exemplar system for analysis of fibrillin microfibrils due to their amenability for isolating microfibrils in a pure form [23], [24].

Therefore, we aimed to investigate on the macroscale how the zonule fibers in the ciliary zonule are organized into bundles, on the microscale how the zonule fibers are constructed from fibrillin microfibrils, and on the nanoscale how the fibrillin microfibril is composed of fibrillin molecules. To address each level of hierarchy, different imaging techniques were used. Serial block-face scanning electron microscopy (SBF-SEM) imaging and thick-section electron tomography were used in an integrated manner between macro- and microscales to create three-dimensional (3D) reconstructions of the bovine ciliary zonule to interrogate zonule fiber and microfibril bundle structures, respectively. These approaches were coupled with negative-stain transmission electron microscopy (TEM) with single-particle averaging of tissue-purified microfibrils to generate a nanoscale 3D model of the fibrillin microfibril repeating unit. This approach has allowed the modeling of individual microfibrils and their organization in fibers within the zonule, and which covers length scales across 5 orders of magnitude has the potential to be applied to other hierarchical tissue structures.

## Results

### Organization of ciliary zonule fibers

**Fig. 1:**
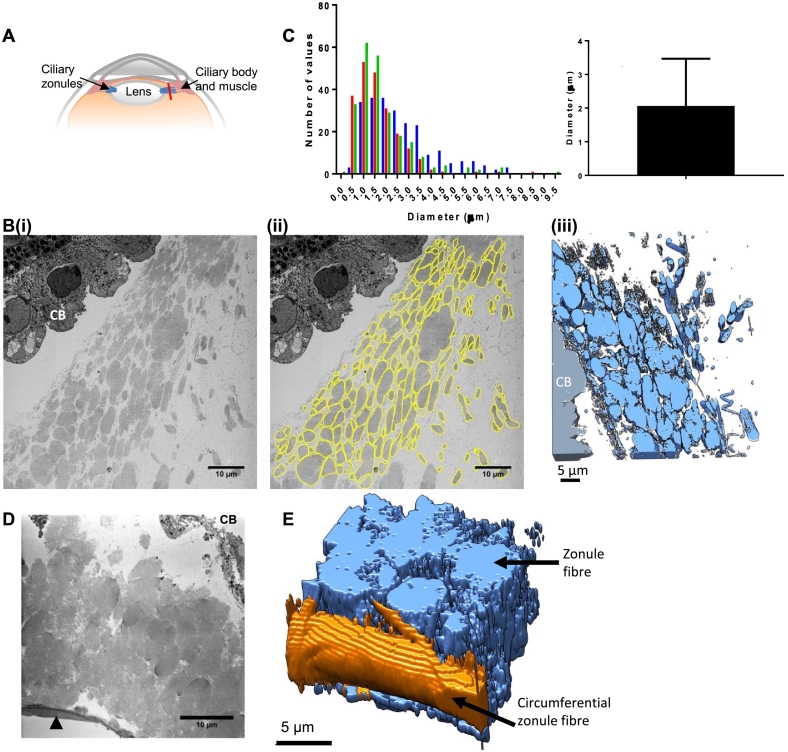
Bundles of ciliary zonule fibers are wrapped together. Bovine ciliary zonules were imaged using SBF-SEM to reveal the structure of the ciliary zonule fibers. (A) A schematic diagram of the eye is shown. The cilary zonules project radially connecting the lens capsule to the ciliary body. The ciliary zonules were sectioned along the sagittal plane of the eye taking transverse cross sections as indicated by the red line. (B) A representative image from an SBF-SEM data set from a region close to the ciliary body is shown. The ciliary body is in the top left corner of the image and indicated by CB. Different-sized zonule fibers can be seen which have been contoured in yellow in panel ii. The scale bar represents 10 μm. A subvolume of this data set was rendered using Chimera and tilted shown in panel iii to view down the zonule fiber axis. The scale bar represents 5 μm. (C) A histogram of zonule fiber diameters. Diameters from three SBF-SEM data sets from three animals were measured in ImageJ and shown in red, blue and green (*N* = ~ 230 for each data set). Most fiber diameters were in the range 0.5 to 3.5 μm, and the mean diameter was 2.06 μm ± 1.40 (S.D.) shown in the histogram (*N* = 683). (D) A number of zonule fibers were seen running perpendicular to the fiber bundles. In the bottom left corner, one zonule fiber runs perpendicular to the other fibers (indicated by a black arrowhead). The scale bar represents 10 μm. (E) A subvolume of this data set was rendered. A circumferentially organized ciliary zonule fiber is shown in orange. The scale bar represents 5 μm.

To determine how fibrillin microfibrils are organized into ciliary zonule fibers, the ciliary zonule was dissected from bovine ocular tissue and the ciliary apparatus (spanning from the ciliary body to the lens capsule; Fig. 1A) was fixed, stained and embedded for imaging by SBF-SEM. In this technique, the surface of the tissue sample is imaged using scanning electron microscopy (SEM) before an inbuilt microtome removes a section from the tissue block. This allows for the serial imaging of a large volume of tissue which can be reconstructed into a 3D volume without intensive realignment and processing of images. Sections of ~ 100 nm in thickness were removed from the surface of the sample block between scans (SI Movie 1), and three data sets of 1500 slices were collected.

**Fig. 2:**
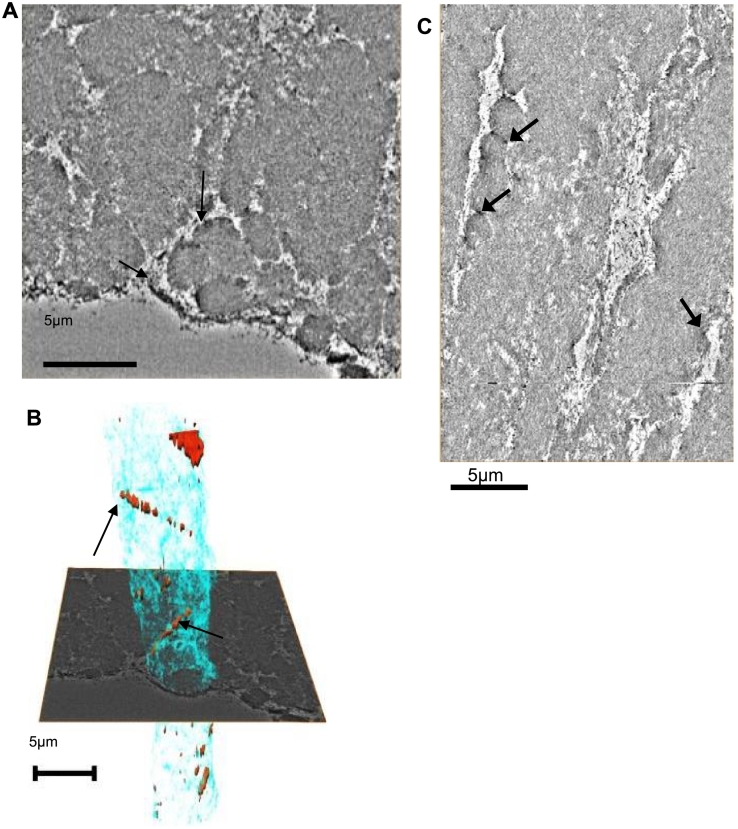
Parallel zonule fibers are stabilized by interweaving strands. (A) Higher-magnification image of zonule fibers shows individual zonule fibers bounded by darkly stained regions at the perimeters of the fibers (indicated by black arrows). Inspection of the 3D volume shows that these regions correspond to smaller fibers wrapping around the zonule fibers, echoing the larger-scale organization shown in Fig. 1. (B) A 3D rendering of a segmented ciliary zonule fiber is shown in blue embedded in a slice of the SBF-SEM volume. More electron-dense fibers around the perimeter are shown in red and highlighted with black arrows. The volume was segmented using Amira. (C) Virtual slice from the SBF-SEM data set shown orthogonal to the image in panel A. Two zonule fibers run vertically in the image with denser patches at their periphery (black arrows), which are the smaller fibers seen wrapping the larger fibers in panel A. The scale bars represent 5 μm.

In a region close to the basement membrane of the non-pigmented ciliary body epithelium, the ciliary zonule was composed of both well-defined ciliary zonule fibers and amorphous fibrillar masses (Fig. 1B). Although in a single two-dimensional (2D) image, the individual fibers are not clearly defined, in the serial sections as shown in SI Movie 1, the bundles are more easily discerned. As the contrast difference between zonule fibers was low, fibers could not be automatically segmented so the diameters of zonule fibers were manually measured in ImageJ taking measurements every 100 sections. The fiber diameters mainly ranged from 0.5 to 3.5 μm and the mean diameter was 2.06 ± 1.40 μm (S.D.); a histogram of the fiber diameter size distribution is shown in Fig. 1C. The zonule fibers generally run parallel to the axis of the ciliary zonule; however, circumferentially arranged fibers were also seen wrapping around the perimeter of bundles of aligned zonular fibers (Fig. 1D and E; SI Movie 2). Furthermore, individual zonule fibers had electron-dense material defining their perimeter (Fig. 2), which on inspection of the 3D volumes was revealed as smaller fibers weaving between and wrapping around individual zonule fibers (Fig. 2B; Movie 3), echoing the macroscale packing arrangement. These denser patches were further investigated using electron tomography to determine whether the microfibril spacing or packing was different.

### Microfibril–microfibril and microfibril–basement membrane contacts

**Fig. 3:**
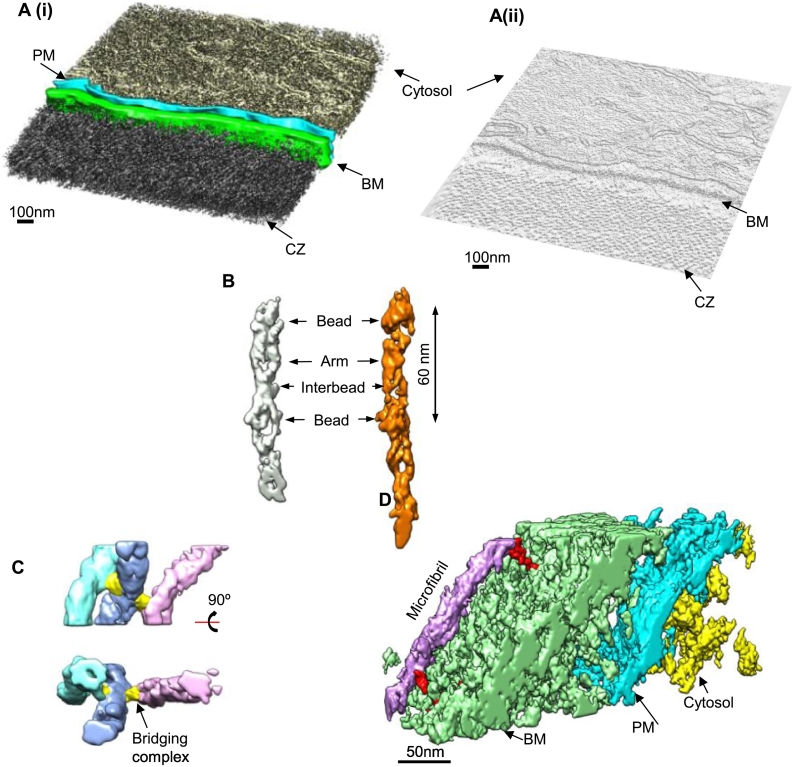
Fibrillin microfibril connections within the zonule. (A) (i) A tomogram of a region of the ciliary zonule adjacent to the basement membrane (BM) of the non-pigmented ciliary epithelium is shown rendered and colored according to features. PM = plasma membrane. The scale bar represents 100 nm. (ii) A 2D slice from the tomogram shown in Ai. (B) Individual fibrillin microfibril volumes were computationally extracted from tomograms and rendered in UCSF Chimera. The bead, arm and interbead regions, which have been described previously for extracted microfibrils [12], can be identified. (C) From the tomograms, microfibril contacts could be observed. Sub-tomogram volumes show bridging molecules mediating lateral associations, which have been highlighted in yellow. (D) A volume from the tomogram showing a microfibril contacting the basement membrane. Direct contacts between the microfibril and the basement membrane are highlighted in red.

To investigate the microstructure and composition of the zonule bundles, the same tissue blocks were sectioned for electron tomography. Thick sections of ~ 250 nm were cut, parallel to the block face imaged by SBF-SEM (with the ciliary zonule in transverse cross section). Sections were imaged using an FEI G2 Polara TEM operating at an accelerating voltage of 300 kV. Low-magnification images are shown in SI Fig. 1. Areas of interest were selected, and tilt series were collected from + 65° to − 65° at a magnification of 23,000 ×. Tilt series were aligned and tomograms were reconstructed using weighted back projection in IMOD. A representative tomogram collected on a region close to the basement membrane is shown in Fig. 3A. Microfibrils are highly aligned in this region, as previously described [25], but are not parallel with the basement membrane. With this approach, individual microfibrils could be resolved within the ciliary zonule. Individual microfibril volumes were extracted from the tomograms and showed a very similar structure to tissue-purified microfibrils [12] with characteristic bead, arm and interbead regions (Fig. 3B). Within the tomograms, contacts could be seen between microfibrils. The contacts were frequently situated at the bead regions of the microfibrils and appeared to be mediated by additional density, perhaps showing the presence of bridging proteins (Fig. 3C; SI Fig. 2). The bead regions are thought to be composed of the N- and C-terminal regions of fibrillin-1. Many microfibril-associated proteins, such as fibulins, LTBPs and A Disintegrin and Metalloproteinase with Thrombospondin motif (ADAMTS) and ADAMTS-like proteins have been shown *in vitro* to bind to the N-terminal region of fibrillin-1 [26], [27], [28], [29] and could be contributing to the tissue packing here. The tomogram could also be interrogated to analyze contacts with the basement membrane of the non-pigmented ciliary body epithelium [30]. Figure 3D shows one example of a microfibril which is at a different angle to the main bundle and runs parallel to the ciliary body making multiple contacts along the length of the microfibril, suggesting how interactions with the basement membrane may be mediated. Fibrillin colocalizes with perlecan in this basement membrane [30], and perlecan could be one of a number of proteins that mediate these interactions.

### Directionality and packing within ciliary zonule microfibril bundles

**Fig. 4:**
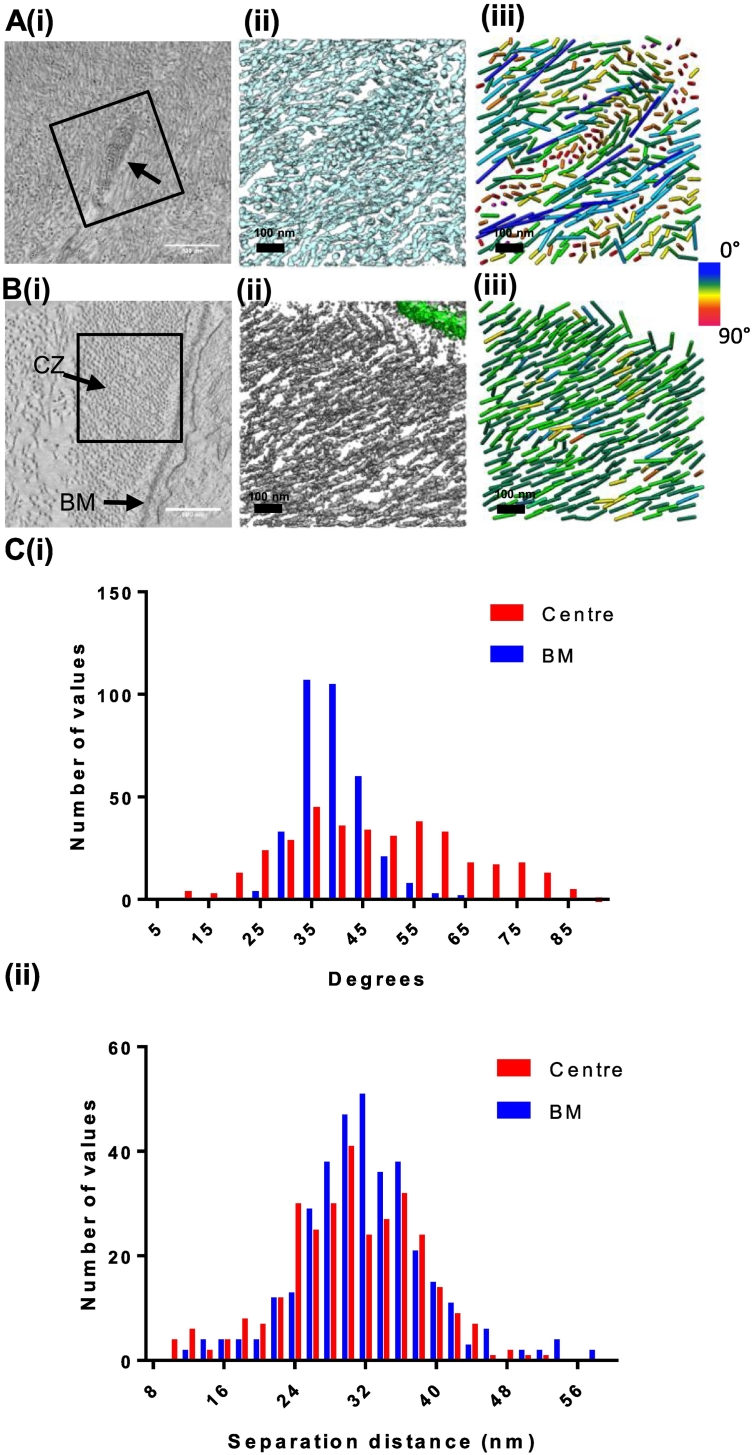
Correlation of microfibril orientation and packing with tissue location. Representative slices from tomogram volumes of (Ai) a microfibril bundle in the center of the ciliary zonule and (Bi) adjacent to the basement membrane of the non-pigmented ciliary body epithelium are shown. 3D rendering of these regions of the tomograms is shown in panels Aii and Bii. Microfibrils within these sub-tomograms (Aii and Bii) were tracked and colored according to their angle relative to neighboring microfibrils and shown in panels Aiii and Biii. The number of microfibrils in each volume was similar, *N* = 343 microfibrils for volume (Aii) and *N* = 362 for volume (Bii). The microfibril tracking is also represented in graphical form as the relative microfibril orientation (Ci) and the separation distances between microfibrils (Cii). These measurements were repeated for a second ciliary zonule sample and shown in Supplementary Fig. 3.

To investigate microfibril packing in different regions of the ciliary zonule, including the electron-dense areas at the periphery of zonule fibers observed by SBF-SEM (Fig. 2), sub-tomogram volumes were extracted from a region toward the center of the ciliary zonule containing an electron-dense region (Fig. 4A) and a second region adjacent to the basement membrane of the non-pigmented ciliary body epithelium (Fig. 4B; SI Fig. 1). The microfibrils in the tomograms were tracked and colored according to their relative angle using IMOD. Histograms of the relative microfibril orientations in these two regions showed that the distribution of orientations of microfibrils differed between the area in the center of a ciliary zonule fiber and the region close to the ciliary body (Fig. 4C(i); SI Fig. 3)). The center of the ciliary zonule fiber was composed of microfibrils in a meshwork-like arrangement, whereas closer to the basement membrane, the microfibrils were more highly aligned in parallel bundles. Both regions, however, had a mean distance between the centers of microfibrils of 30 nm, showing that the electron-dense region was not more densely packed (Fig. 4C(ii)) and the difference in staining was due to microfibril orientation differences in this region. Reconciling these data with the SBF-SEM images, this electron-dense region was a bundle of microfibrils running perpendicular to and wrapping around the zonule fiber.

### 3D structure of the fibrillin microfibril repeating unit

**Fig. 5:**
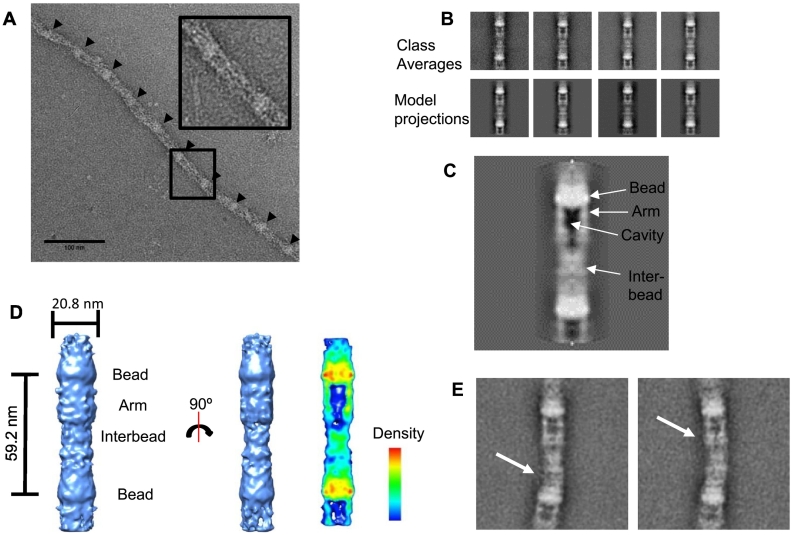
Fibrillin microfibrils have a tubular structure with hollow cavity. (A) An image of a fibrillin microfibril purified from the ciliary zonule is shown. Arrows highlight the bead regions of the microfibril. The scale bar represents 100 nm. Inset: one microfibril repeat region magnified. (B) The top panel shows class averages of the aligned particles, and the bottom panel shows 2D projections of the final reconstructed 3D model shown in panel D. (C) A slice through the center of the rotational average of the 3D model shown in panel D with regions of the model labeled. (D) A 3D model of the fibrillin repeat with regions of the model labeled. The model has 2-fold symmetry imposed along the microfibril axis. The right panel shows a bisected map of the fibrillin model, with red representing areas with the highest density. (E) Reference-free class averages of aligned microfibril repeats show areas of flexibility highlighted by white arrows. For panels B, C and E, the box size is 102 × 102 nm.

To determine the 3D structure of the fibrillin microfibril repeat, microfibrils were purified from ciliary zonules and imaged using negative-stain TEM (Fig. 5A). Microfibrils had the characteristic beads-on-a-string appearance, and ~ 3000 microfibril repeating units were extracted from the images (Fig. 5B). An initial cylindrical model was generated using SPIDER [31] and iteratively refined against the data by projection matching using FindEM [32] and SPIDER procedures (similar to as described in Ref. [33]) to create a final 3D microfibril model (Fig. 5D). Two-fold symmetry was applied along the microfibril axis as models would iteratively degrade during refinement when symmetry was removed (data not shown). The class averages of the aligned particles were very similar to the 2D projections of the 3D model (Fig. 5B), suggesting that the final model is representative of the data. The resolution of the microfibril model was estimated using Fourier shell correlation (FSC) at the 0.5 threshold to give an estimated resolution of 43 Å (SI Fig. 4).

The class averages and rotational average of the 3D model reveal the distinct asymmetrical fibrillin banding pattern with clearly defined bead, arm and interbead regions (Fig. 5C), as described in previous 2D analyses of microfibril structure [12]. Some heterogeneity is apparent in the class averages due to flexibility in the microfibrils along the microfibril axis. The largest movement is in regions adjacent to the bead, highlighted by white arrows in the class averages shown in Fig. 5E and further supported by the diffuseness of the interbead region in class averages where alignment is localized to the bead region (as seen in SI Fig. 5A). This flexibility observed along the length of the microfibril could be responsible for the degradation of the microfibril model when imposed symmetry was relaxed during refinement. The reconstructed model has a bead-to-bead periodicity of 59.2 nm and has a diameter of 20.8 nm at its widest point (Fig. 5D), which is similar to previously published measurements of microfibril dimensions [24]. The 3D model has a hollow tube-like structure that is consistent with observations of microfibrils using quick freeze deep etch (QFDE)-EM [11]. If the 3D model is viewed in cross section, the bead is prominent along with an internal cavity which are details seen in the 2D images. Previously, the features seen in 2D images were difficult to reconcile with the cylindrical appearance seen with QFDE. However, if we consider that the 2D TEM images are a projection of the 3D microfibril, this explains how these two observations can be reconciled, since the QFDE images provide a pseudo-3D image.

### Pseudo-symmetry within regions of the microfibril

**Fig. 6:**
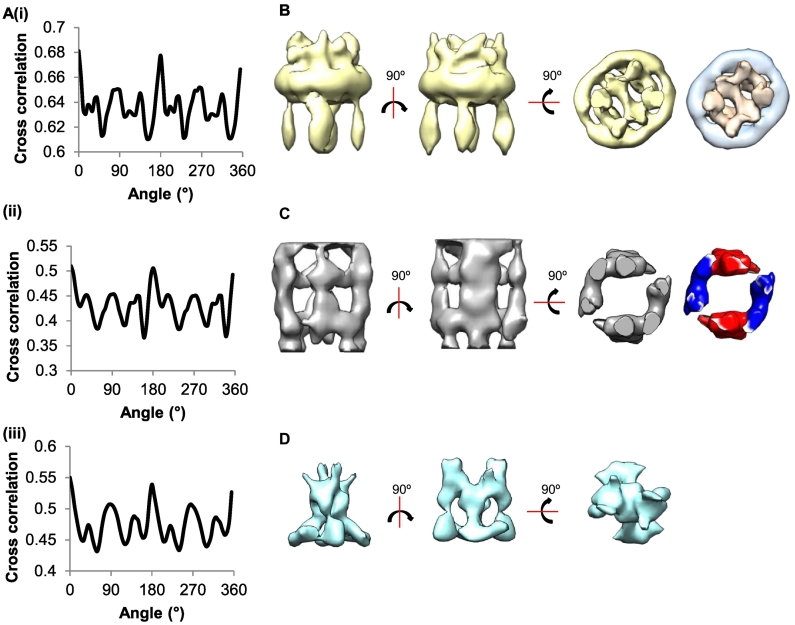
Regions within the fibrillin microfibril have symmetrical features. The bead, arm and interbead regions of the microfibril were refined separately using region-specific masking. Rotational cross-correlation graphs of the sub-models regions for the bead (Ai), arm (Aii) and interbead (Aiii) regions against 0° model projections. The correlations reveal higher-order pseudo-symmetries within the microfibril. Consequently, the bead (B), arm (C) and interbead (D) regions were further refined applying 2-fold symmetry along the microfibril axis. In panel B, the right-hand panel shows the core bead structure at higher threshold showing the outer ring (blue) and central core (pink). The right-hand panel in panel C shows the four arms colored blue and red according to the symmetric pairs.

To overcome potential heterogeneity caused by the flexibility of microfibrils and with the objective of increasing the resolution of the microfibril reconstruction, separate sub-models of specific microfibril regions (i.e., the bead, arm and interbead regions) were created by refining the initial cylindrical starting model of the full microfibril (i.e., no symmetry applied). Sub-models were created by aligning particles to these specific regions using binary masks. The reconstructions were then projected at 5° increments and cross-correlated against the initial data set using FindEM. All the microfibril regions showed a strong correlation at 180°, suggesting that all regions of fibrillin microfibrils have two-fold symmetry along the microfibril axis and further correlation at 90° and 45° suggesting 4- and 8-fold pseudo-symmetry in some regions of the microfibril (Fig. 6A). This information about microfibril composition and the structure of these different regions with 4- and 8-fold symmetries supports the suggestion that the microfibril consists of eight fibrillin microfibrils arranged into four dimers [12]. Two-fold symmetry was therefore imposed along the microfibril axis for subsequent rounds of refinement.

### Sub-modeling fibrillin microfibril regions overcomes flexibility and increases model resolution

The model of the bead region has a complex arrangement of fibrillin molecules with two distinct features, a central core and outer ring structure. Using segmentation in UCSF Chimera, the bead can be separated into the bead core region and the ring region for clarity (Fig. 6B). If the model is rendered at a threshold of 40% of the estimated volume, the core structure can be seen. The core of the bead forms a complex interwoven structure that can be seen to connect to the arm regions. When the arm region of the microfibril is refined, the increase in detail shows that this region comprises four distinct arms (Fig. 6C). The arms emerge from the bead region, bow out and re-join to meet in the interbead region. The arms were segmented into separate volumes, but as 2-fold symmetry is applied along the microfibril, the four arms can be categorised into two symmetric pairs.

Separate refinement of the interbead region revealed a structure with three distinct features (Fig. 6D). At the top, four separate strands connect to the arm region. The central layer has a more compact structure that splits into four globular densities at the bottom of the structure. Class sum and projection images show that areas outside the bead region have become very poorly defined (SI Fig. 5). The regions adjacent to the interbead region appear to have lost definition in the averages, which suggests that the interbead region is relatively rigid and the surrounding regions are areas of flexibility in the microfibril.

### Eight symmetrically arranged fibrillin monomers can be accommodated in the interbead region

**Fig. 7:**
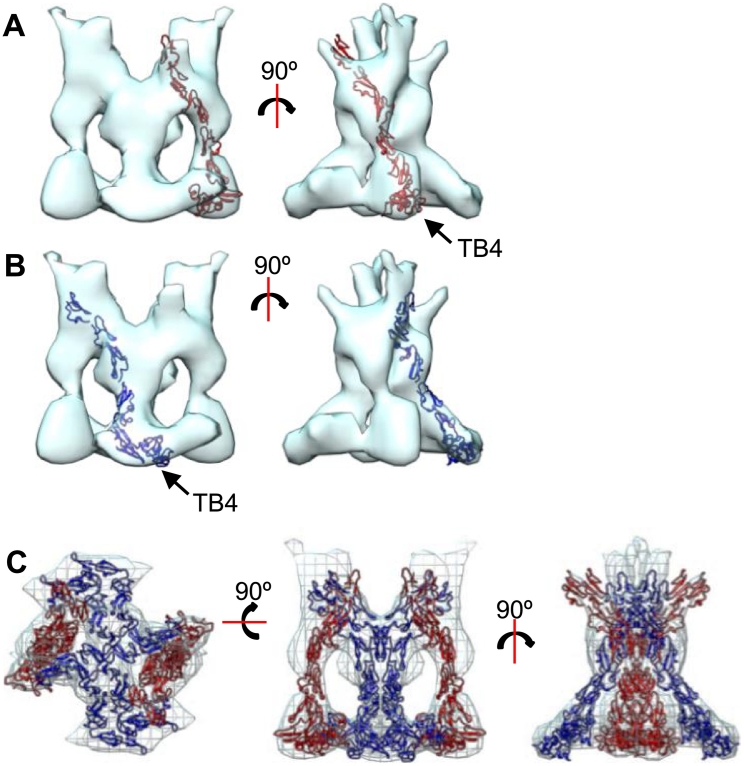
Modeling symmetrically arranged fibrillin monomers into the interbead region. Crystal structures of six cbEGF domains and a TB domain were docked into the interbead region using UCSF Chimera. The interbead structure has 2-fold symmetry imposed along the microfibril axis. Therefore, domains were modeled into the EM density in symmetric pairs shown in red (A) and blue (B). Domain models fitted into the structure in pairs of dimers with a total of eight molecules shown (C).

The 3D reconstructions of the arm and interbead regions show very well-defined structure with a clear symmetric arrangement. Although only 2-fold symmetry was applied during the data processing, four clearly delineated strands can be visualized (Fig. 7A). Although the resolution of the 3D reconstruction was too low to accurately dock or identify specific fibrillin domain locations, modeling was used to see whether these strands could accommodate single fibrillin chains or dimers. Six cbEGF domains and one TB domain (arbitrarily representing cbEGF17-TB4 using the structure of cbEGF22-TB4 (1UZQ) [34] and homology models of cbEGF17–21 [9]) were modeled into each non-symmetrised strand in the interbead reconstruction using UCSF Chimera (Fig. 7A and B). In each strand, one fibrillin molecule left some density unoccupied; therefore, eight fibrillin chains were modeled into this region (Fig. 7C). Modeling of these chains into this region suggests that the fibrillin molecules take a non-linear path through the region, with a TB domain forming the more globular base of the interbead. Furthermore, the volume of this region supports the presence of eight fibrillin molecules within each microfibril repeating unit.

## Discussion

Fibrillin is an evolutionarily ancient elastic molecule that provides long-range elasticity to mammalian elastic tissues and is essential for the extracellular regulation of growth factor signalling. We wanted to understand how a single molecular building block is used to construct elastic tissues with nano- to macroscales of hierarchical structure to provide these unique functional properties. Therefore, to determine how individual fibrillin microfibrils are arranged in higher-order structures, bovine ciliary zonules were imaged using SBF-SEM and electron tomography. We show that parallel zonule fibers are encircled by circumferential wrapping fibers, and although microfibrils are highly aligned in the region close to the basement membrane, microfibril bundles are not parallel to the basement membrane. We show individual microfibrils peeling off from the bundle to run in parallel and make multiple contacts with the basement membrane. Microfibrils also make contacts with each other and binding proteins, and we show that these contacts predominantly occur at the bead region of microfibrils. Our 3D reconstructions of purified microfibrils confirm that microfibrils contain a central cavity and reveal that it is only found in the interbead region, which explains the light/dark appearance of microfibrils in tissue section images. We also show that throughout all regions of the microfibril (i.e., bead, interbead and shoulder), there is a pseudo-8-fold symmetric arrangement of fibrillin molecules.

**Fig. 8:**
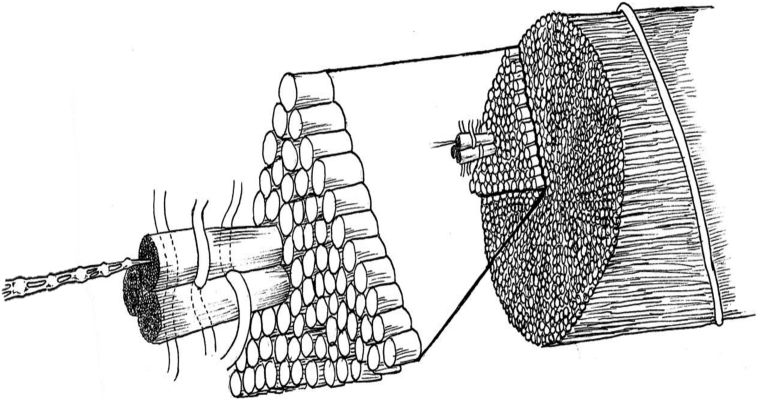
Packing model for the ciliary zonule. A sketch of the ciliary zonule hierarchical structure (left to right) where fibrillin molecules form zonular fibers. Individual zonule fibers and fiber bundles are wrapped by circumferentially arranged fibers of different diameters. Zonule fibers have interweaving wrapping fibers.

Ciliary zonule fibers were roughly cylindrical and consistent with sizes observed previously [18]. Zonular fibers were predominantly organized along the axis of the zonule but with some fibers circumferentially organized, taking a helical path encircling and wrapping the parallel fibers (Fig. 1D). A previous study of fibrillin-1-stained ciliary processes showed transverse striations running across the zonular fibers, which are consistent with these circumferentially arranged fibers [35]. Around the circumference of individual zonule fibers, electron-dense regions were observed (Fig. 2). When rendered in 3D, it was apparent that these regions were smaller diameter fibers wrapping around each zonule fiber in a manner that echoed the gross packing arrangement. These appear to be fibrillin microfibrils having the recognizable beaded repeating structure and same diameter. Although LTBP2 is also present in the ciliary zonules, colocalizes with fibrillin in zonular fibers and is essential for the normal function of the ciliary zonules [36], LTBP2 is not a core component of microfibrils [37] and thin fibers formed by LTBP1 are smaller and without a beaded structure [10]. The wrapping of both fiber bundles and individual fibers may stabilize their structure and connect fibers allowing them to perform their mechanical role (Fig. 8).

Using electron tomography, the same tissue blocks were imaged following SBF-SEM. Tomograms revealed that there was a range of microfibril diameters in the zonule with a mean diameter of 11.4 nm, similar to tissue microfibril measurements [1] but smaller than the bead diameter of 20 nm for purified microfibrils. However, the tomogram measurements are made on different cross sections through the microfibril (i.e., the bead, interbead or arm regions) and as such represent an average microfibril diameter. The average periodicity was 60 nm (SI Fig. 6), and the average distance between the center of microfibrils was 28 nm, which correlates with the spacing between microfibrils in tensioned ciliary zonules measured by X-ray diffraction [38]. Lateral associations between microfibrils and bridging molecules were observed between microfibrils, predominantly found at the bead region, which may help to maintain the packing of microfibrils in ciliary zonule. Small filaments connecting adjacent microfibrils have been reported previously in ciliary zonule microfibrils [11]. Recent studies of murine models of ectopia lentis have shown that LTBP-2 plays an essential role in the maintenance of ciliary zonule fiber stability [36] and could therefore be a candidate for forming links between microfibrils. In addition, disruption of ADAMTSL4, also expressed in the eye, leads to progressive disruption of the ciliary zonule and is another candidate linking protein [39]. LTBP-2 binds to the N-terminus of fibrillin [40], which is predicted to be located in the microfibril bead region [24], [41], and although there are currently no data on the precise binding site for ADAMTSL4, related ADAMTS-like proteins also bind to the N-terminal region of fibrillin [29].

The microfibrils imaged by electron tomography in the zonule were tubular in appearance, and the bead and arm regions could be identified in some repeats (Fig. 3B). This is consistent with microfibrils imaged by QFDE, which showed tube-like structures where beads could be visualized [11]. Internal microfibril features are seen more prominently in 2D images of purified microfibrils (Fig. 5C), while the 3D structure has a hollow tubular appearance with an internal cavity. Therefore, our data show that the features apparent in images of extracted microfibrils (arms, cavity etc) are due to them being represented in 2D projection, and our 3D reconstructions of both extracted and in situ microfibrils show that previous imaging data can be reconciled with a hollow tube-like structure with internal cavity and beaded features.

Flexibility was observed along the axis of the microfibril (Fig. 5E), resulting in conformational heterogeneity, which was overcome by constructing shorter sub-models of the bead, arm and interbead regions. These shorter models could be refined to higher resolution and more detailed structures determined. The sub-models have a combined volume which is consistent with eight fibrillin molecules per microfibril repeat. Rotational correlation of the sub-models shows that 2-fold symmetry runs along the microfibril axis, which is consistent with observations seen with 2D averaging and tomographic reconstructions [12], [24]. Higher-order symmetries are also present in some segments of the microfibril. The correlation peaks suggest pseudo-4- and 8-fold symmetries, which support the hypothesis that the fibrillin molecule comprises eight molecules per repeat [12], [24].

Example cbEGF domains and a TB domain were modeled into the interbead region of the microfibril, which could be fitted in an extended but non-linear array. At this resolution, it is not possible to determine the location of specific domains, which means that the precise molecular packing within the microfibril cannot be distinguished yet. However, the models show that the fibrillin molecules take a non-linear path through the microfibril repeat. The bead region has been shown by immunolocalization to contain the N- and C-terminal regions of fibrillin. Furthermore, the initial step in microfibril assembly is thought to be head-to-tail interaction between the N- and C-terminal regions of fibrillin and the recombinant C-terminal half of the fibrillin molecule can assemble to give bead-like structures [42]. Here we show that the bead has a complex structure with interwoven core, encased by an outer ring, which could represent the N-terminal region encased by the C-terminal region. Future work using cryoEM imaging approaches should allow resolution of the precise molecular packing within the microfibril.

Here we used SBF-SEM imaging and thick-section electron tomography on the same tissue block to bridge the macro- and micro-scales. The combination of these techniques has broad applicability to samples containing structural information spanning multiple length scales including other ocular tissues, tendons and the vasculature. Furthermore, since microfibrils could be purified from zonules, we coupled these approaches with negative-stain TEM to span 6 orders of magnitude from nanometer to millimeter, enabling the modeling of individual microfibrils in the zonule and their organization into bundles and fibers (Fig. 8). These data will allow us to analyze differences in the zonule organization with fibrillin mutations to better understand how this gives rise to ectopia lentis.

## Materials and Methods

### SBF-SEM and electron tomography

Ciliary zonules were dissected from bovine eyes and prepared for SBF-SEM and electron tomography as previously described [43]. Bovine ciliary zonule samples were oriented so the ciliary zonule was imaged in transverse cross section by SBF-SEM using a Quanta FEG 250 (FEI) equipped with a Gatan 3View ultramicrotome at 3.8 kV. Back-scattered electron images were collected following the automated removal of a 100-nm slice. Data sets of 1500 images were collected at 14 nm/pixel from three different animals. A depth of 150 μm of tissue was imaged, and the data were rendered with Amira 6.3.0 (Thermo Scientific) and UCSF Chimera [44]. Individual zonule fibers with defined perimeter were manually segmented in ImageJ [45] by fitting ellipses to their contours to measure the minor diameters, to account for the plane of the section [43]. For electron tomography, thick sections (~ 250 nm) were cut from samples using a Diatome diamond knife and Leica ultramicrotome. Sample sections were mounted on formvar carbon-coated copper slot grids (Agar Scientific), and 10-nm colloidal gold solution was applied to both sides. Single-axis tilt series were taken at 23,000 × from − 65° to + 65° in 1 ° steps using a FEI Tecnai G2 Polara TEM at 300 kV. Images were collected using a Gatan Ultrascan 4000 CCD camera and the software SerialEM [46]. Tomograms were generated by weighted-back projection in IMOD using the Etomo workflow [47]. Individual microfibrils were traced though tomograms using IMOD to calculate their relative orientation. Analysis of microfibril diameter and spacing was carried out using ImageJ using the particle analysis tool [45].

### Negative-stain TEM and single-particle averaging

Fibrillin microfibrils were purified for negative-stain TEM as previously described [23] and imaged under low-dose conditions at − 0.5- to − 1-μm defocus at 21,000 × (4 Å/pixel) on a Tietz TVIPs F214A CCD camera using a FEI Tecnai 12 twin TEM at 120 kV. A total of 2797 microfibril particles were manually boxed using EMAN2 [48], with a box size of 512 × 512 pixels. Particles were CTF corrected by phase-flipping before edge-mean normalization and band-pass filtering. A high-pass filter of 600 Å and a low-pass filter of 20 Å were applied using a top-hat filter, using SPIDER [31]. Particle stacks were iteratively rotationally and translationally aligned, using the local projection matching program FindEM [32]. The modified version of FindEM for particle stacks is available at the CCPEM website (www.ccpem.ac.uk). The initial template was an average image of the unaligned stack of particles. An initial 3D model was constructed by creating a cylindrical average model from the sum average of the aligned particle set, using SPIDER, filtered to 20 Å; this was subsequently refined using a projection matching approach as we described for collagen VI microfibrils [49]. To evaluate the reconstructions, projections were compared non-quantitatively to class sum images created with a non-reference based classification method using IMAGIC5 [50]. Sub-models were generated by alignment of the bead, arm and interbead regions using binary template masks in FindEM specific for these regions. The resolution of the final models was estimated using FSC at the 0.5 FSC threshold for two models reconstructed from two halves of the data using SPIDER.

The following are the supplementary data related to this article.

Supplementary figures Image 1

Movie 1 Serial block-face SEM data set of the ciliary zonule. A movie showing a representative sub-set of the SBF-SEM image stack analyzed in Fig. 1D. Movie 1

Movie 2 3D rendering of ciliary zonule fibers. The movie shows 2D slices through the SBF-SEM data set shown in Fig. 1D before it is rendered in 3D. Individual ciliary zonules can be seen with circumferentially wrapped fibers around the perimeter. The contrast of the data has been inverted. Movie 2

Movie 3 3D segmentation of an individual ciliary zonule fiber. A movie of a 3D rendered segmented ciliary zonule fiber. The ciliary zonule fiber is shown in blue, and the more electron-dense fiber around the perimeter is shown in orange. A 2D orthogonal slice of the SBF-SEM data is also shown. Movie 3
