# Supplementary material for: Multiscale Imaging Reveals the Hierarchical Organization of Fibrillin Microfibrils
Source: J Mol Biol. 2018 Oct 19;430(21):4142–55. doi: 10.1016/j.jmb.2018.08.012 (PMC6193142; doi:10.1016/j.jmb.2018.08.012)
Supplement: Supplementary file 1 — Supplementary figures [file mmc1.pdf]

Supplementary Figure 1

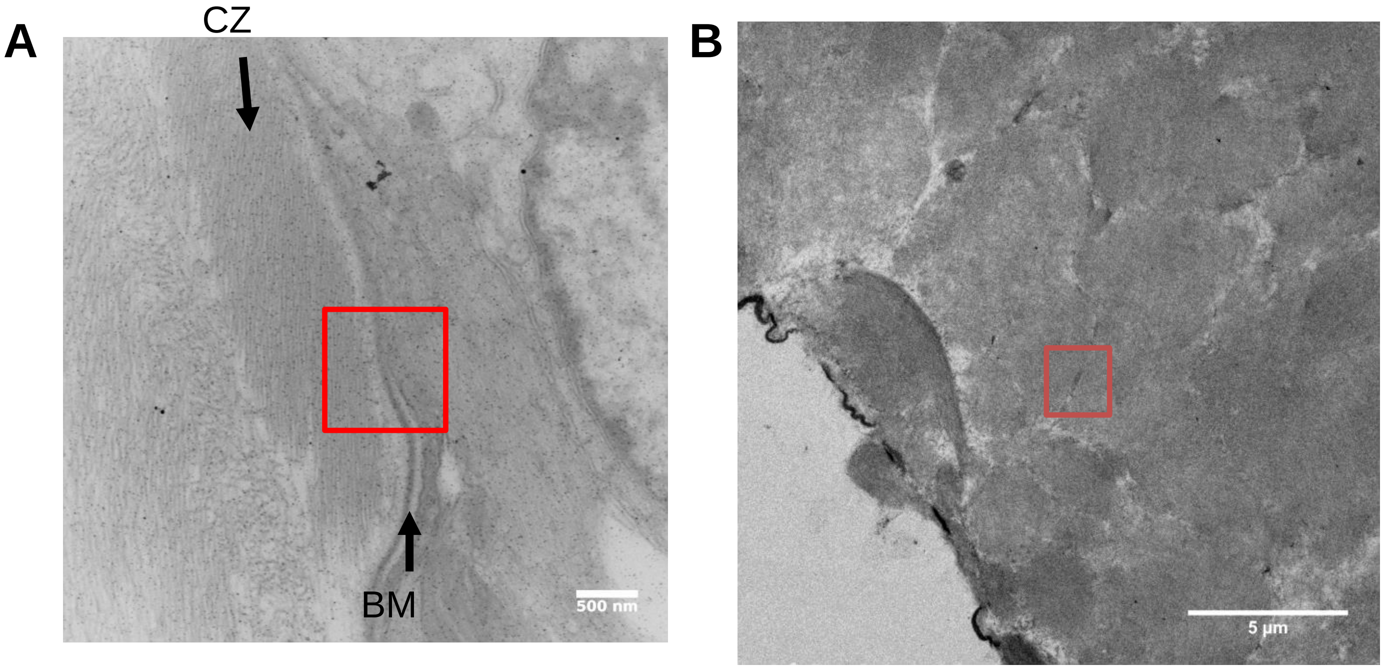

Supplementary Figure 1: A) TEM image of bovine ciliary zonule (CZ) adjacent to the basement membrane (BM) of the ciliary body. Scale bar = 500 nm. (B) TEM image of a region near the centre of the ciliary zonule with a denser region at the periphery of a zonule fibre. Scale bar = 5 µm. The areas highlighted in red were collected as tomograms.

Supplementary Figure 2

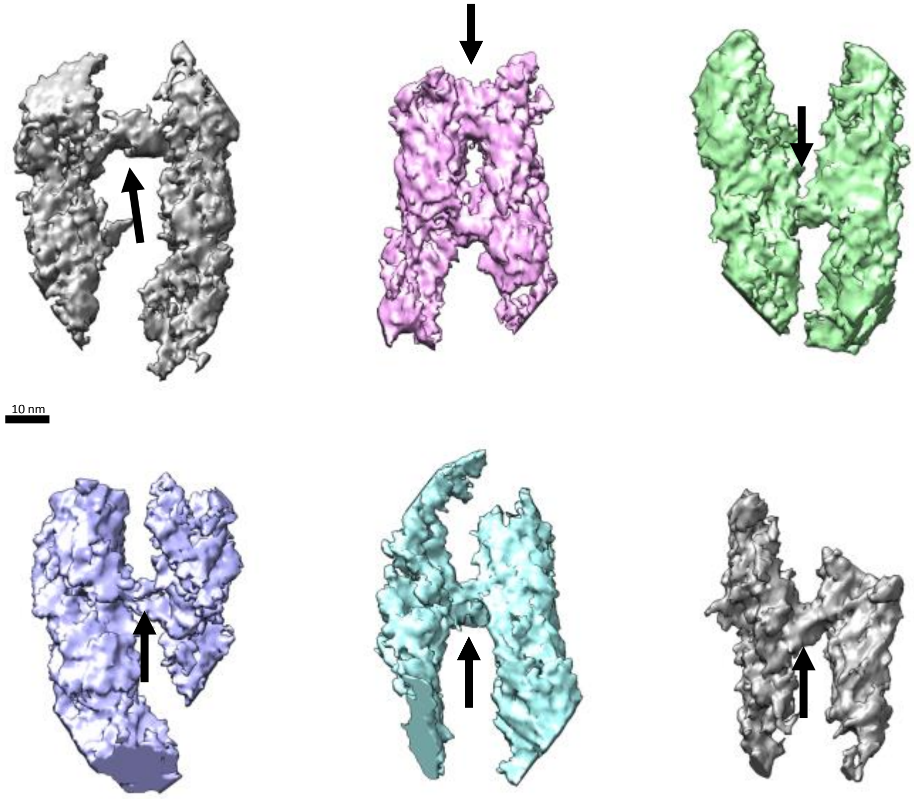

Supplementary Figure 2: Representative extracted sub-tomogram volumes of microfibril contacts. Microfibril bridging molecules are indicated by black arrows. Volumes were rendered using UCSF Chimera.

Supplementary Figure 3

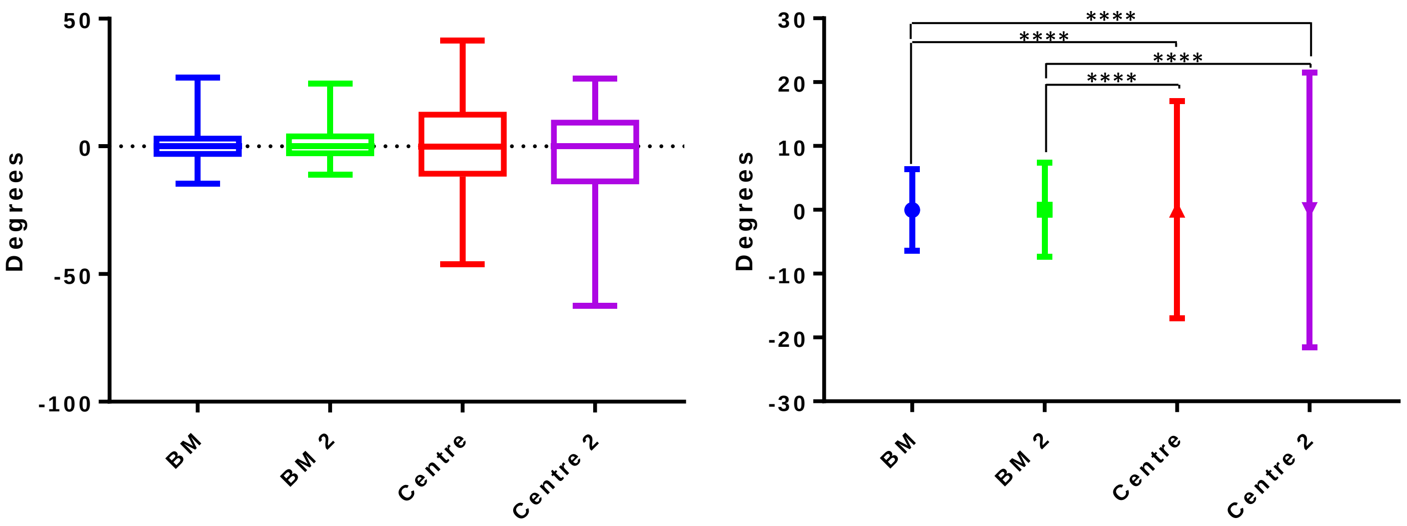

Supplementary Figure 3: (A) Box and whisker plot of the normalised angle between microfibrils close to the basement membrane (BM) and at the centre of the ciliary zonule showing that the microfibrils at the centre of the ciliary zonule have a wider range of orientations than the more aligned microfibrils near the basement membrane. The box shows the median and 25th to 75th percentile and the whiskers show the minimum to maximum range of the data. (B) The mean angle and standard deviation is plotted for the same data. The variance in the range of microfibril orientation at the basement membrane is significantly different to that at the centre of the zonule. **** P-value ≤0.0001, F test to compare two variances. Data is shown for four tomograms (two animals for each area), the number of microfibrils measured in each volume was similar ~N=350 for each dataset, and data were normalised to their median values for comparison between datasets. The data from BM and Centre are also shown in Fig. 4A and B.

Supplementary Figure 4

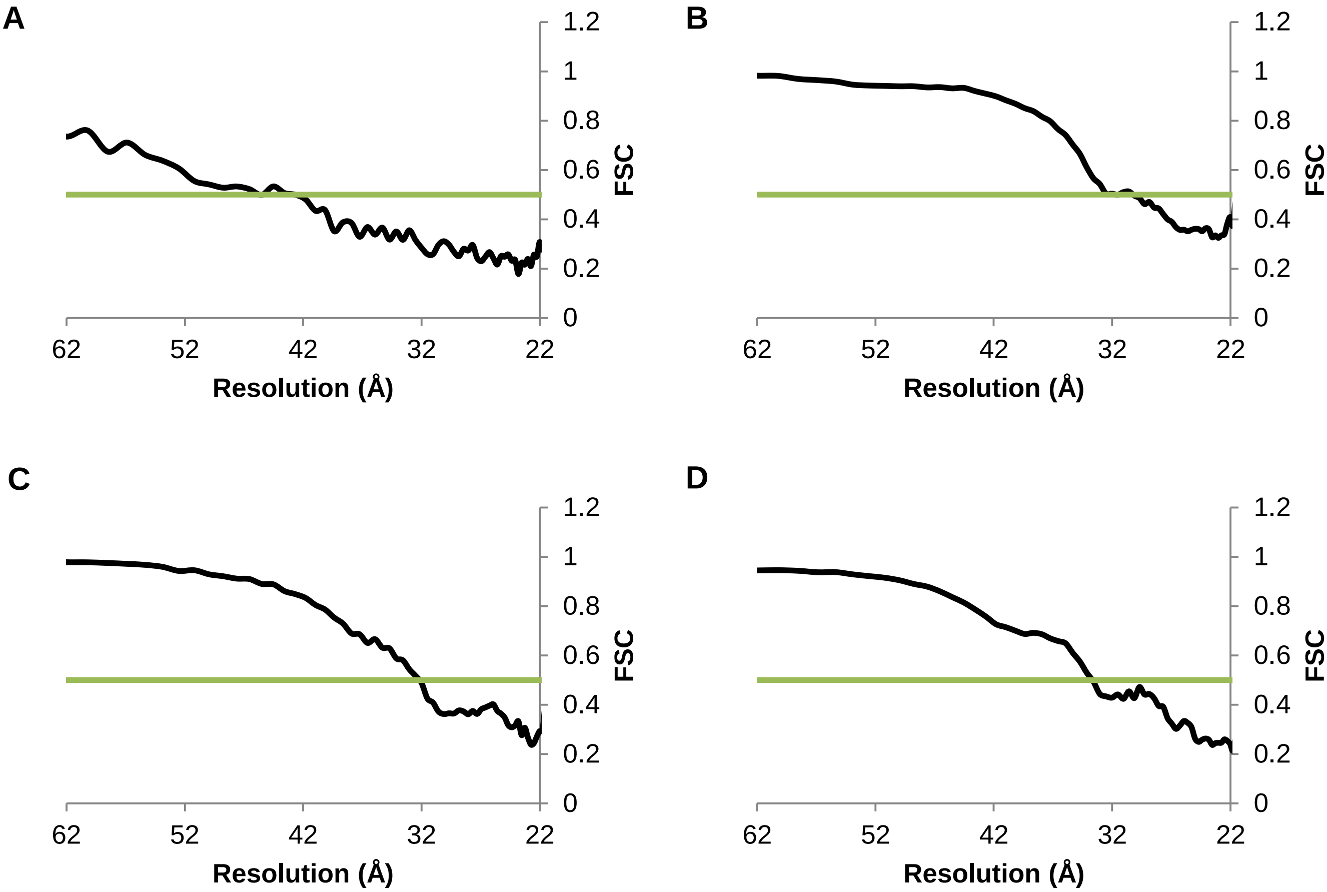

Supplementary Figure 4: Fourier shell comparison of microfibril reconstructions. The FSC curves for (A) the full microfibril reconstruction, and the sub-models of the (B) bead, (C) arm and (D) interbead regions were plotted against spatial resolution. The green line shows the 0.5 threshold for resolution estimation.

Supplementary Figure 5

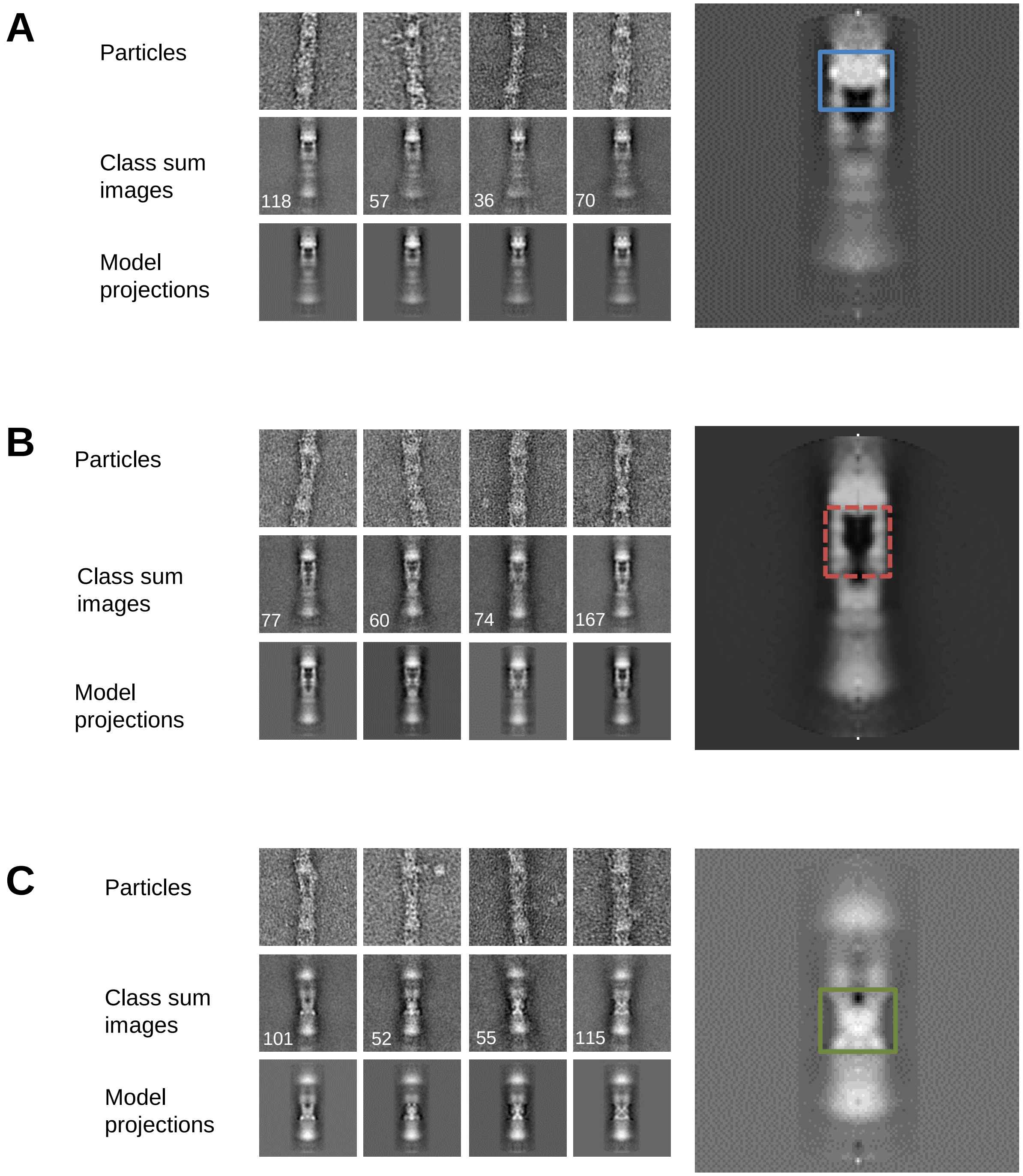

Supplementary Figure 5: Microfibril sub-region reconstructions. The (A) bead, (B) arm and (C) interbead regions were reconstructed separately to increase the resolution of these areas of the microfibril reconstruction. In (A-C) The top panel shows aligned particles extracted from negative stain TEM images. The middle panel shows class sum images of the aligned particles and the bottom panel are 2D projections around the fibre axis of the final reconstructed 3D volume. The right panel is a slice through the centre of the rotational average of the 3D reconstruction; the different areas which were refined are highlighted with coloured boxes. The box sizes of the images are 102 x 102 nm. The number of particles which contribute to each class is shown in white.

Supplementary Figure 6

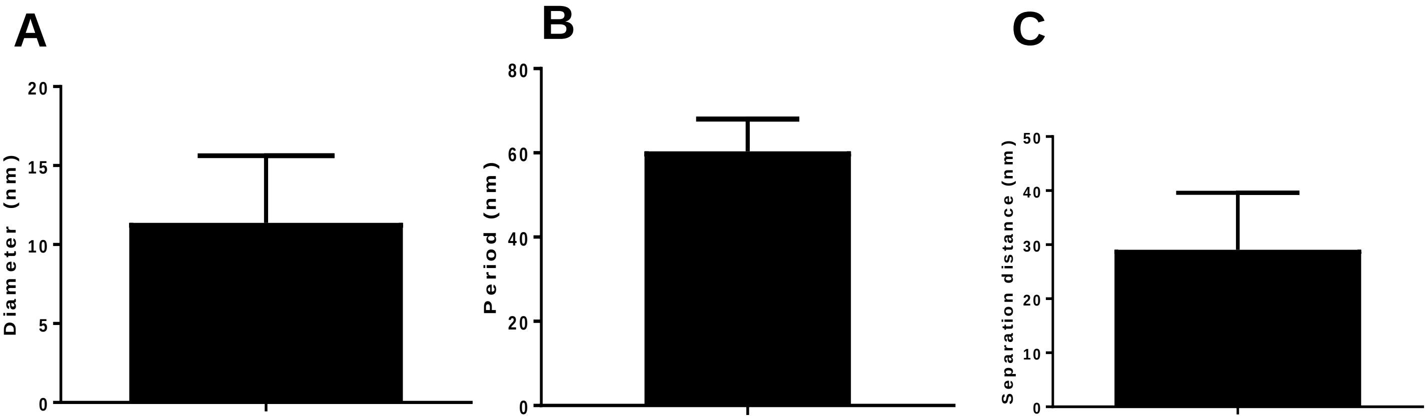

Supplementary Figure 6: Analysis of microfibril parameters. (A) The ImageJ particle analysis tool was used to measure individual microfibril diameters. The mean diameter was 11.4 nm ± 4.46 S.D. N=7990. The diameters range from 2 to 24 nm. (B) Microfibril periodicity was measured in UCSF Chimera using the volume tracer tool. The mean periodicity was 60.4 nm +/- 7.94 S.D. N=63. (C) Microfibril separation distance was 28 nm +/- 10.43 S.D. N=4838. Measurements on diameter and separation distance were taken from four representative tomograms (two animals with two tomograms from each), including the two shown in Fig. 4 and periodicity data from three tomograms.
